# Indocyanine green as a near-infrared theranostic agent for ferroptosis and apoptosis-based, photothermal, and photodynamic cancer therapy

**DOI:** 10.3389/fmolb.2022.1045885

**Published:** 2022-12-07

**Authors:** Hsiang-Ching Tseng, Chan-Yen Kuo, Wei-Ting Liao, Te-Sen Chou, Jong-Kai Hsiao

**Affiliations:** ^1^ Department of Medical Imaging, Taipei Tzu Chi General Hospital, Buddhist Tzu-Chi Medical Foundation, New Taipei City, Taiwan; ^2^ Department of Research, Taipei Tzu Chi General Hospital, Buddhist Tzu-Chi Medical Foundation, New Taipei City, Taiwan; ^3^ School of Medicine, Tzu Chi University, Hualien, Taiwan

**Keywords:** indocyanine green, organic anion transmembrane polypeptide, near-infrared, theranostic effect, apoptosis, ferroptosis

## Abstract

Ferroptosis is a recently discovered programmed cell death pathway initiated by reactive oxygen species (ROS). Cancer cells can escape ferroptosis, and strategies to promote cancer treatment are crucial. Indocyanine green (ICG) is a near-infrared (NIR) fluorescent molecule used in the imaging of residual tumor removal during surgery. Growing attention has been paid to the anticancer potential of ICG-NIR irradiation by inducing ROS production and theranostic effects. Organic anion transmembrane polypeptide (OATP) 1B3 is responsible for ICG metabolism. Additionally, the overexpression of OATP1B3 has been reported in several cancers. However, whether ICG combined with NIR exposure can cause ferroptosis remains unknown and the concept of treating OATP1B3-expressing cells with ICG-NIR irradiation has not been validated. We then used ICG as a theranostic molecule and an OATP1B3-transfected fibrosarcoma cell line, HT-1080 (HT-1080-OATP1B3), as a cell model. The HT-1080-OATP1B3 cell could promote the uptake of ICG into the cytoplasm. We observed that the HT-1080-OATP1B3 cells treated with ICG and exposed to 808-nm laser irradiation underwent apoptosis, as indicated by a reduction in mitochondrial membrane potential, and upregulation of cleaved Caspase-3 and Bax but downregulation of Bcl-2 expression. Moreover, lipid ROS production and consequent ferroptosis and hyperthermic effect were noted after ICG and laser administration. Finally, *in vivo* study findings also revealed that ICG with 808-nm laser irradiation has a significant effect on cancer suppression. ICG is a theranostic molecule that exerts synchronous apoptosis, ferroptosis, and hyperthermia effects and thus can be used in cancer treatment. Our findings may facilitate the development of treatment modalities for chemo-resistant cancers.

## 1 Introduction

Indocyanine green (ICG), an infrared fluorescent dye, has been widely used in clinical settings to determine liver function before liver resection and to visualize the retinal vasculature for decades (Lu and Hsiao, 2021). Recently, ICG has been employed for the *in vivo* imaging of tumors because of its near-infrared (NIR) fluorescent capability that enables deep tissue penetration and cancer detection (Wu et al., 2018; Wu et al., 2019a; Wu et al., 2019b). Moreover, studies have reported the feasibility of using ICG, in combination with NIR phototherapy that produces reactive oxygen species (ROS) with sequential apoptosis, for the detection of hepato-cellular carcinoma (Radzi et al., 2012; Shirata et al., 2017). A study indicated that ICG potentiates the photothermal effect (Porcu et al., 2016). Due to its safety, antitumor properties, and deep tissue penetration ability, ICG has the potential to be used as an anticancer molecule.

Our previous study reported ICG is metabolized by organic anion transporting polypeptide 1B3 (OATP1B3) (Wu et al., 2018). OATP1B3 regulates anion transportation into the cytoplasm and is physiologically expressed in the liver (Shitara, 2011). Furthermore, OATP1B3 is highly expressed in various cancer cells (Imai et al., 2013). However, the prognostic role of OATP1B3 in cancer remains unclear. Therefore, targeting OATP1B3-expressing cancer cells by ICG, observing these tumor cells through imaging modalities, and killing them can be a beneficial cancer treatment strategy.

Fibrosarcoma is an uncommon malignancy that has a poor response toward chemotherapy (Donato Di Paola and Nielsen, 2002). Apart from complete resection, the eradication of residual fibrosarcoma by chemotherapy alone is difficult. Only 4%–11% of patients with advanced fibrosarcoma benefitted from chemotherapy (Augsburger et al., 2017). Hence, a new strategy for treating fibrosarcoma is required. HT-1080 cells originated from patients with fibrosarcoma have been examined extensively (Rasheed et al., 1974; Daigeler et al., 2008; Hsiao et al., 2013; Wu et al., 2018). Although OATP1B3 has a restricted expression toward liver, we previously transfected HT-1080 cells with OATP1B3, which successfully transported ICG into the cytoplasm (Wu et al., 2018). In this study, we evaluated the photothermal and photodynamic effects of ICG and their benefits for treating chemo-resistant cancer by using HT-1080 cells overexpressing OATP1B3.

Ferroptosis is an iron-dependent, programmed cell death mechanism that occurs after exposure to excess reactive oxygen species (Dixon et al., 2012; Liang et al., 2019; Su et al., 2020). Ferroptosis is entirely different from apoptosis, necrosis, and autophagy (Ye et al., 2020). Cancer cells resistant to apoptosis after chemotherapy might be vulnerable to ferroptosis (Xu et al., 2019). Strategies inducing ferroptosis in cancer cells have been evaluated. Studies have reported the efficacy of some anti-cancer drugs, such as sorafenib and erastin, in promoting ferroptosis in both the cancer cell culture and tumor xenograft (Dixon et al., 2012; Lachaier et al., 2014). ICG, in combination with infrared irradiation, was reported to be effective in generating reactive oxygen species in cell culture (Wang et al., 2019a). Nevertheless, the feasibility of using ICG and infrared irradiation to induce ferroptosis for treating cancer has not been examined. This study examined the feasibility of using ICG and infrared irradiation to treat fibrosarcoma cells and elucidated underlying cell death pathways. The findings of this study can facilitate the development of novel cancer therapies.

## 2 Materials and methods

### 2.1 Cell line and culture

HT-1080 cells were cultured in Dulbecco’s modified Eagle’s medium (DMEM) (Thermo Fisher Scientific, Waltham, MA, United States) supplemented with 10% fetal bovine serum (FBS) (Biologic Industries, Cromwell, CT, United States), 100 U/mL of penicillin, and 100 μg/ml of streptomycin (Invitrogen, Carlsbad, CA, United State). A stable cell line, OATP1B3-expressing HT-1080 cells were constructed through lentivirus transduction as described previously (Wu et al., 2018). All the cells were maintained in a humidified atmosphere of 5% CO_2_ at 37°C and passaged with 0.5% trypsin (Thermo Fisher Scientific) for 1.5 min at 80%–90% confluence.

### 2.2 Cellular uptake of ICG

Crystalline ICG (molecular weight of ICG sodium iodide, 924.9 g/mol) was purchased from Sigma Aldrich (St. Louis, MO, United States). A 2 mM ICG stock solution in water was prepared and separated into several aliquots and stored at −20°C. The ICG signal detection was performed using both a microplate reader and fluorescence microscope. To evaluate the transportability of ICG by using SpectraMax M5 system (Molecular Devices, Sunnyvale, CA, United States), 2.5 × 10^4^ cells were seeded in 96-well plates for 1 day before the addition of 20 and 200 μg/ml ICG for 2 h. Excess ICG was removed by washing the cells three times with phosphate-buffered saline (PBS). The intensity signal was measured at the excitation and emission wavelengths of 780 and 830 nm, respectively, under shaking for 2 s and normalized to that of the control group. In addition, we visualized the ICG signal by using a fluorescence microscope (Leica Microsystems DMI6000 B, Wetzlar, Germany). A 49030-ET-ICG filter cube (Chroma Technology, Olching, Germany) was chosen to detect the intensity of ICG. In total, 5 × 10^5^ cells were seeded in six-well plates for 1 day before 50 μg/ml ICG was added for distinct time points. Then, the cells were washed three times with PBS after ICG incubation and fixed with 4% formaldehyde. Finally, the slides were mounted in SlowFade Gold Antifade Reagent with 4,6-diamidino-2-phenylindole (DAPI) (Thermo Fisher Scientific).

### 2.3 *In vitro* PDT

The OATP1B3-expressing cells were seeded in 96-multiwell microplates at 2.5 × 10^4^ cells/100 μl medium/well. The cells were divided into four groups: control (without ICG treatment and laser irradiation), ICG only (without laser irradiation), laser only (without ICG treatment), and PDT (with both ICG treatment and laser irradiation). The supernatant of the cells treated with ICG for 4 h was removed, and the cells were carefully washed with PBS to eliminate any remaining dye. Finally, each well was covered with a drug-free medium. Homogeneous irradiation was successively performed using laser diodes emitting light at 808 nm with a maximum optical output power of 15 W. The effects of laser irradiation with different fluence rates and times were examined. The irradiation conditions were as follows: One of the fluence rates was adjusted to 0.098 W/cm^2^, and the energy density was 54 J/cm^2^. The other fluence rate was adjusted to 0.2 W/cm^2^, and the energy density was 60 J/cm^2^. The laser diodes were set 1 cm below the microplate. In addition, a second NIR irradiation was conducted 24 h after the first irradiation, and each well was replaced with fresh medium and incubated for 0, 24, 48, and 72 h.

### 2.4 Reactive oxygen species detection

To evaluate ROS synthesis, DCFH-DA was employed to examine intracellular ROS generation. Briefly, the cells exposed to ICG and NIR irradiation were incubated with 10 μM DCFH-DA in 200 μl DMEM for 30 min at 37°C. Subsequently, the cells were washed three times with PBS. For the positive group, the cells were treated with 10 μM H_2_O_2_ for 2 h before DCFH-DA treatment. All data were acquired either using a fluorescence reader or a fluorescent cell imager (ZOE, Bio-Rad, Hercules, CA, United States). To investigate the lipid ROS levels of these cells, the cells were incubated with 2 μM C11-BODIPY 581/591 (Thermo Fisher Scientific, Waltham, MA, United States) in a culture medium for 1 h and then washed with PBS. After trypsinization, the cells were harvested and processed for flow cytometry (BD Bioscience, San Jose, CA, United States) at an excitation wavelength of 488 nm combined with an emission wavelength of 517–527 nm.

### 2.5 Mitochondrial membrane potential measurement

The apoptosis signaling pathway is triggered when cells are under a toxic or an unfavorable environment. MMP decreases during apoptosis. Therefore, MMP can serve as an early indicator of cell apoptosis. The method used for MMP measurement was based on a previous study (Smiley et al., 1991). The cells were washed with PBS after treatment with ICG and NIR laser irradiation. The cells were then incubated with the JC-1 dye (Thermo Fisher Scientific) at 37°C for 30 min in a cell culture incubator. Finally, the fluorescence intensity was analyzed using a fluorescence microscope and a fluorescence spectrometer. The cells treated with 50 μM carbonyl cyanide 3-chlorophenylhydrazone (CCCP) were used as positive controls because CCCP is a common MMP disruptor.

### 2.6 Annexin V and propidium iodide staining

After distinct treatment (group1: control; group2: ICG only; group3: laser only; group4: PDT), cells were harvested and washed twice in cold PBS, and resuspended in annexin V-FITC and PI (Elabscience Biotechnology Inc., Houston, TX, USA) for 30 min in the dark. Cells were then measured with a flow cytometer (BD Bioscience, San Jose, CA, United States) equipped with an air-cooled argon laser that emitted at 488 nm. Data from at least 10^4^ cells were analyzed with FlowJo software.

### 2.7 Thermal observation and imaging analysis

In brief, 200 μl of sample solutions containing different ICG concentrations were added into 96-well plates and exposed to an 808-nm laser for different time courses. After ICG-NIR treatment, thermal images were captured using an IR FlexCam Thermal Imager *TI55* (FLUKE, Everett, WA, United States). The HT-1080-OATP1B3 cells treated with ICG but not exposed to 808-nm laser were used as a negative control. All images were analyzed using the open-source image processing software (Fluke Connect) downloaded from the website (https://www.fluke.com/en-us/products/fluke-software/connect). The temperature values for quantification were circled within the edge of the thermal signal.

### 2.8 Cell viability assay

Viability was examined using the MTT assay. MTT is reduced to purple formazan in living cells by mitochondrial reductase. The morphology of the cells in the control, ICG, NIR laser, and PDT groups was examined using a phase-contrast microscope (Eclipse TS100; Nikon, Tokyo, Japan). Then, the MTT reagent was added to each well with the medium at a final concentration of 0.5 mg/ml. After incubation at 37°C for 4 h under a 5% CO_2_ atmosphere, the medium was aspirated from 96-well plates carefully and substituted with an equal volume of solubilizer buffer (DMSO) to dissolve the formazan crystals. The absorbance of the formazan product was evaluated at a wave-length of 570 nm by using a fluorescence plate reader. The results are indicated as the percentage of data obtained with control cultures.

### 2.9 Western blotting

A total of 1 × 10^6^ cells were harvested from six-well plates and lysed using the following steps. The cells were collected, washed with PBS, and lysed using RIPA lysis buffer (Pierce, Rockford, IL, United States) containing 1% Sigma protease cocktail for 30 min at 4°C. The lysates were centrifuged at 12,000 × g at 4°C to obtain solubilized cellular proteins. The protein concentration was measured using a bicinchoninic acid (BCA) protein assay (Pierce, Rockford, IL, United States). Each lane of 12% SDS-PAGE was loaded with 30 μg of the total protein extract, and the proteins were electrotransferred onto nitrocellulose membranes (Sartorius, Göttingen, Germany). The membranes were blocked with 5% nonfat milk and 1% bovine serum albumin (BSA, Biologic Industries, Cromwell, CT, United States) in tris-buffered saline with Tween 20. Subsequently, the blots were probed with specific primary antibodies against PARP-1 (1:200, Santa Cruz Biotechnology, Dallas, TX, United States), Caspase-3 (1:1000, Cell Signaling, Danvers, MA, United States), Bim (1:1000, Cell Signaling, Danvers, MA, USA), Bcl-2 (1:1000, Cell Signaling, Danvers, MA, United States), Bax (1:1000, Cell Signaling, Danvers, MA, USA), GPX4 (1:1000, Cell Signaling, Danvers, MA, United States), SLC7A11 (1:1000, ABclonal, Woburn, MA, United States), and β-actin (1:4000, Cell signaling) separately at 4°C overnight, followed by incubation with HRP-conjugated goat anti-rabbit IgG (1:5000, Zymed, San Francisco, CA, United States) for 1 h at room temperature. The same membrane was reprobed with β-actin as a loading control. Protein bands were detected through enhanced chemiluminescence (Millipore-Sigma, Billerica, MA, United States) by using the BioSpectrum 810 Imaging System (UVP, CA, United States).

### 2.10 Tumor xenografts

Female BALB/cAnN.Cg-Foxnlnu/CrlNarl nude mice (aged 6–8 weeks) were purchased from the National Laboratory Animal Center (Taiwan). A total of 1 × 10^6^ OATP1B3‐expressing HT-1080 cells in 100 μl of DMEM were injected subcutaneously in the bilateral flank. The xenografts were inspected twice a week during the 2 weeks following cell implantation. If a subcutaneous nodule could be visualized, the xenograft size was recorded, and *in vivo* PDT experiments were initiated.

### 2.11 *In vivo* imaging and PDT

After the nude mice were subcutaneously administered the tumor cells for 2 weeks, they were anesthetized with isoflurane and intraperitoneally injected with 10 mg/kg of ICG (solvent: ddH_2_O). Following the administration of ICG, the trend of the ICG signal in the mice was traced through *in vivo* fluorescence imaging at 1, 4, 7, 24, and 28 h post-injection by using the IVIS50 imaging system (Xenogen, Perkin Elmer, MA, United States). The transplanted tumor was illuminated at an excitation wavelength of 780 nm, and fluorescence was obtained using an 845-nm filter. The model mice were prepared as aforementioned for *in vivo* PDT (as shown in Supplementary Figure S2B). Irradiation with a near-infrared laser source (High Power LED driver, THORLABS, Münchner, Germany) was performed 24 h post-injection, and the laser source was placed 1 cm above the xenograft tumor. The fluence rate was 1 W/cm^2^, and the irradiation time was 10 min. Subsequently, the tumor volume was measured every 2 days until day 8. The tumor sizes were calculated using the following formula: [(longest length) × (shortest)^2^]/2 with a digital caliper.

### 2.12 Immunohistochemistry

Tumor tissues were fixed with 10% formalin and were prepared as paraffin-embedded sections (5-μm thick). Slide sections were deparaffinized in xylene (Allegiance Healthcare Corporation, McGaw Park, IL, United StatesA) and then hydrated by passing through graded alcohol to water. Endogenous peroxidase was blocked using 0.3% hydrogen peroxide for 10 min. After a short wash with phosphate-buffered saline with Tween-20 (PBST), the tissue samples were blocked with 5% BSA at room temperature for 1 h. The samples were then incubated with rabbit polyclonal 4 Hydroxynonenal (4-HNE) antibody (dilution 1:200 in 1% BSA; Thermo Fisher Scientific) at 4°C overnight. Furthermore, after a short wash in PBST, the slides were incubated using the EnVision kit (Agilent Technologies Inc., Santa Clara, CA) and were counterstained with hematoxylin. All cover slides were visualized using the ECLIPSE TE2000‐U microscope (Nikon, NY, United States).

### 2.13 Statistics

Each experiment was performed at least in three biological replicates. Data are presented as means ± standard errors (SEM). In addition, statistical analysis was performed using Student’s *t* test, Duncan’s new multiple range tests, and Newman–Keuls and Dunnett’s multiple comparison tests to determine differences (*p < 0.05; **p < 0.01; ***p < 0.001) *via* GraphPad Prism.

## 3 Results

### 3.1 *In vitro* ICG uptake and quenching effect

**FIGURE 1:**
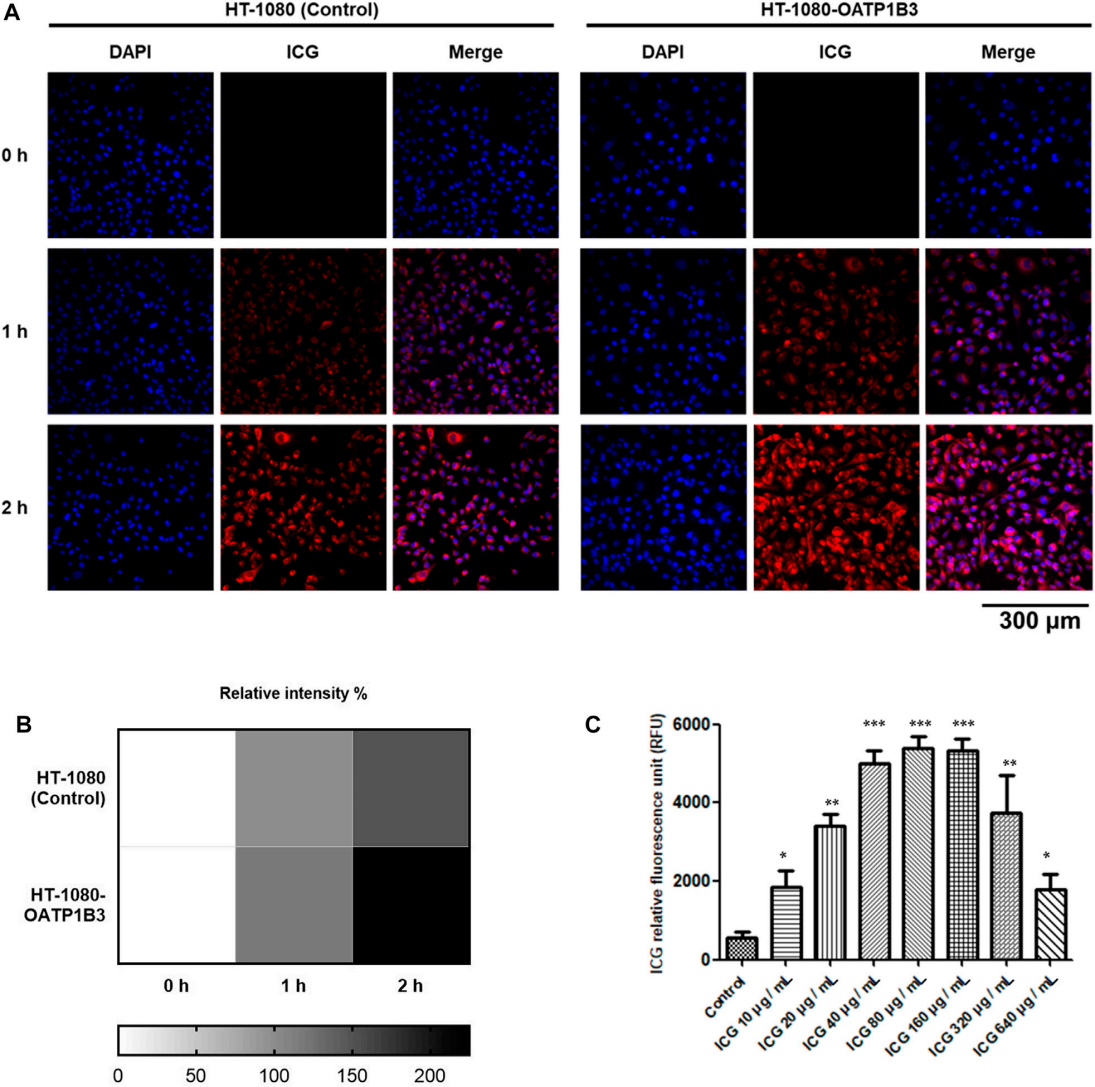
Fluorescence intensity of ICG and quenching effect within cells. **(A)** Fluorescence microscopy performed using a filter cube (49030-ET-ICG) for ICG detection. The cells were treated with the period indicated every 1 h **(B)** Fluorescence intensity of ICG was further quantified (below panel). **(C)** OATP1B3-expressing HT-1080 cells were exposed to various ICG concentrations for up to 4 h. The ICG signal was measured by determining the absorption of ICG at 780 nm and fluorescence emission at 830 nm. Values are indicated as the mean ± standard error of the three replicates of each treatment. Using Student’s *t*-test to analyze our data and *p* < 0.05 was considered significant (*p < 0.05, **p < 0.01, and ***p < 0.001).

Although previous studies have reported that OATP1B3 can uptake ICG (Wu et al., 2018), the quenching effect of ICG within cells has not been addressed in detail. Therefore, we investigated the quenching property of ICG and examined its transportability in OATP1B3-expressing HT-1080 cells. After treating control and OATP1B3-expressing HT-1080 cells with 50 μg/ml of ICG for 0, 1, and 2 h, respectively, the OATP1B3-expressing HT-1080 cells were observed to have a stronger ICG signal than did the control cells, as indicated by fluorescence microscopy findings (Figures 1A). Furthermore, the ICG intensity enhanced with an increase in ICG treatment time. Supplementary Figure S1 presents the flow cytometry results of the uptake of ICG by OATP1B3-expressing HT-1080 cells. To examine the quenching property of ICG within the HT-1080 cells, we treated them with a series of concentrations of ICG and then monitored their relative fluorescence unit by using a spectrometer. The data indicated that the fluorescent signal significantly decreased when the ICG concentration was >160 μg/ml (Figure 1C), which may lead to some responses because the energy emission of ICG interfered at such conditions.

### 3.2 Detection of reactive oxygen species production

**FIGURE 2:**
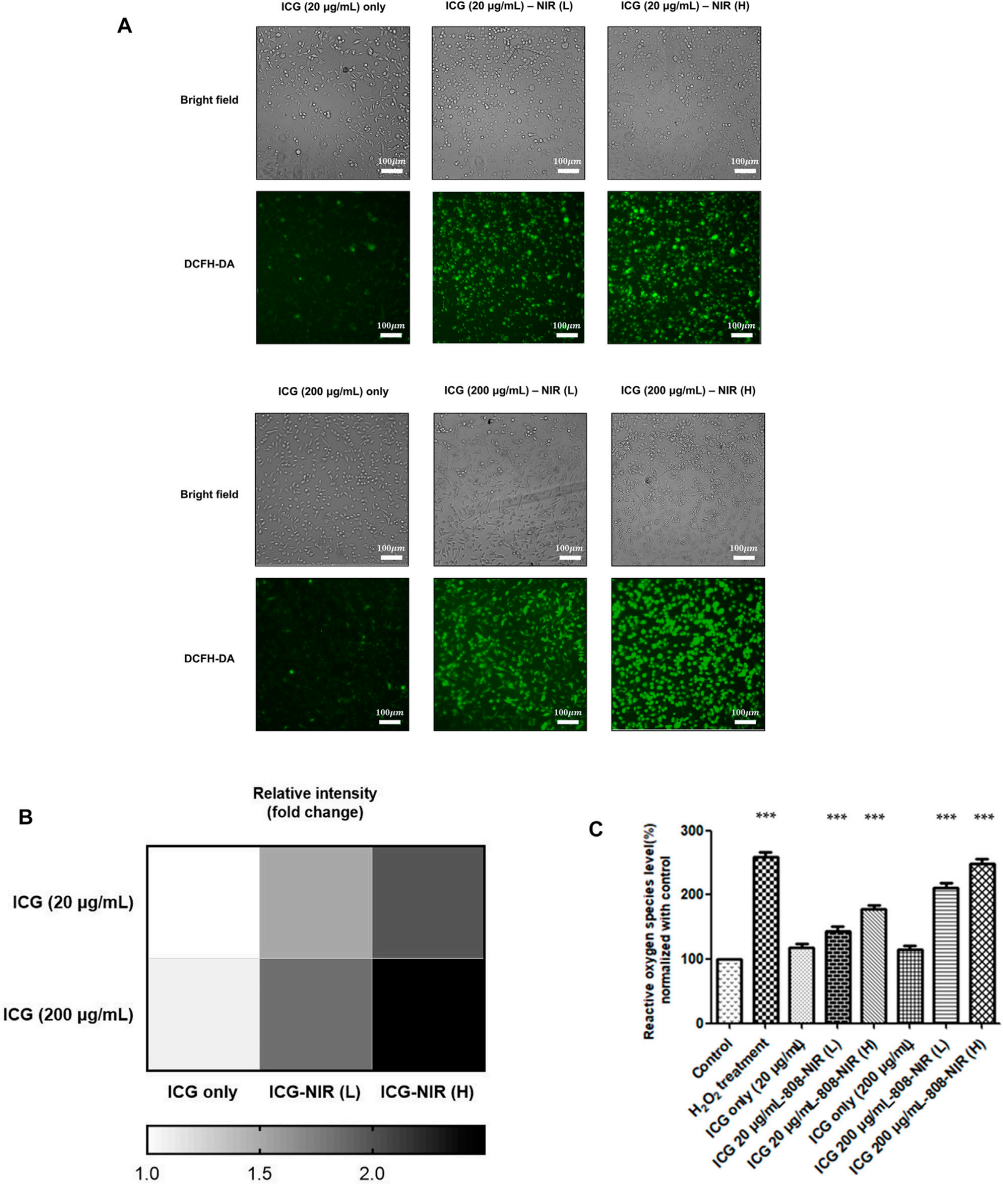
ROS generation after ICG-NIR administration. **(A)** Fluorescence microscopy findings of cells treated with DCFH-DA after ICG-NIR *in vitro*. The bright-field image is depicted for the visualization of the whole-cell culture condition. The cells became spherical after 808-NIR irradiation. **(B)** Fluorescence intensities were further quantified (below panel). **(C)** The relative oxygen species level was analyzed using a fluorescence reader to determine the difference among different experimental conditions. ROS was normalized to the mean of the values of controls and compared between groups by using analysis of variance. Error bars indicate the SEM. ***p < 0.001. L: low-fluence rate; H: high-fluence rate. The scale bar is 100 μm.

To evaluate responses after ICG–NIR administration, we conducted a cytochemical analysis by using 2′,7′-dichlorofluorescein diacetate (DCFH-DA) to examine ROS production. As presented in Figure 2, higher ROS production was observed in the cells treated with ICG–NIR than in the cells treated with ICG alone. Moreover, the cells treated with 200 μg/ml of ICG exhibited markedly enhanced fluorescence intensity under low-fluence or high-fluence laser irradiation. Compared to the 200 ug/mL ICG treated group, the ROS production of the 20 ug/mL ICG treated group is less significant. Additionally, the findings of quantitative analysis indicated that treatment with 200 μg/ml of ICG-NIR considerably increased ROS production, implying that the quenching effect of ICG might be a significant factor contributing to effective photodynamic therapy (PDT). For semi-quantitative determination of the NIR laser effect toward ICG treated cells and for future clinical translation, we did low and high fluence laser irradiation to the ICG treated cells. We found a dose-responsive ROS production effect between the low and high fluence NIR Laser treated group.

### 3.3 ICG-near-infrared exposure reduced mitochondrial membrane potential and induced apoptosis

**FIGURE 3:**
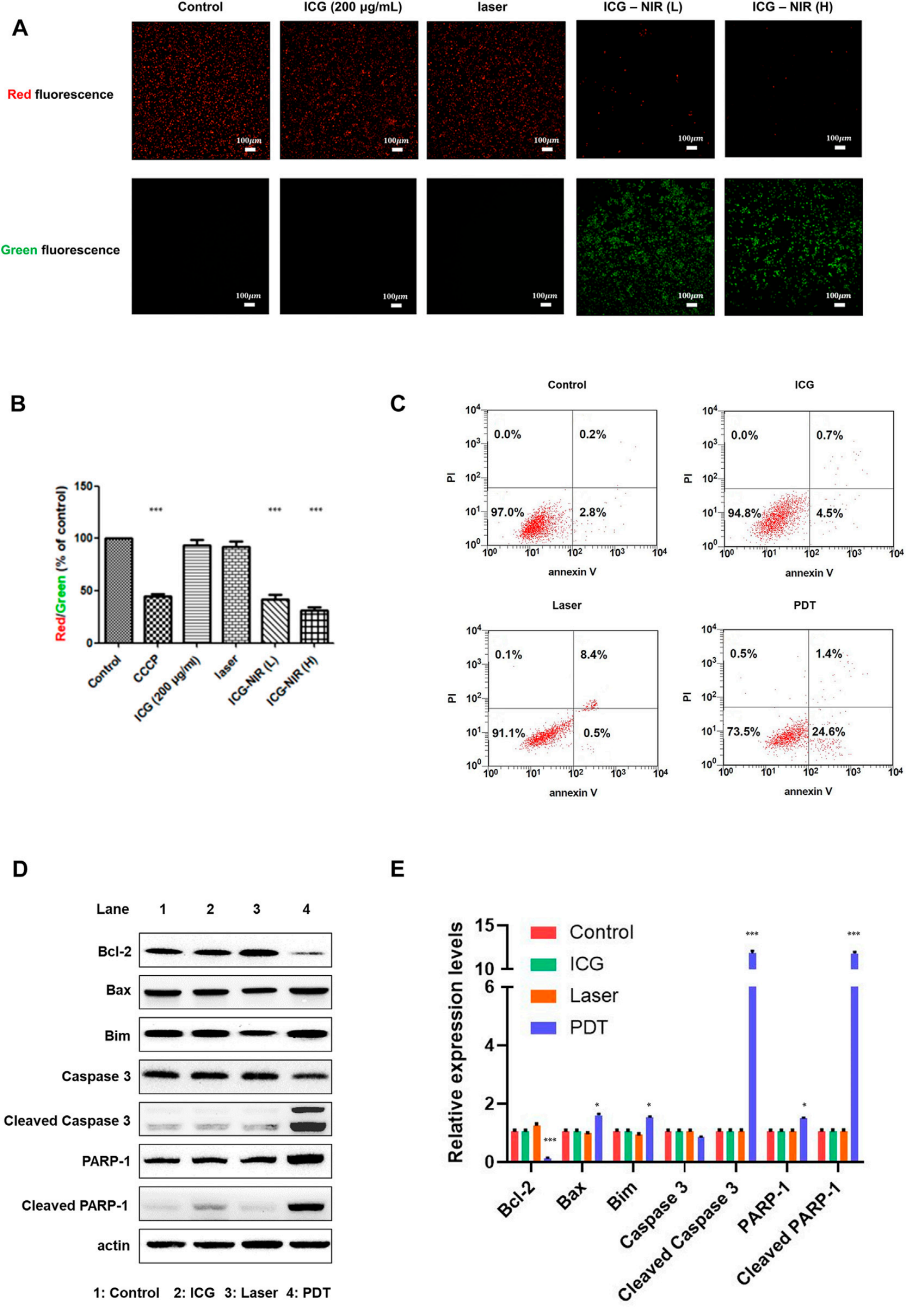
MMP measurement and cell apoptosis analysis post-ICG-NIR administration. **(A)** Fluorescence images of red and green were examined to determine whether mitochondria were healthy after the addition of the JC-1 dye *in vitro*. **(B)** We used a fluorescence reader and calculated the red (−590 nm)/green (−529 nm) fluorescence intensity ratio. Significant MMP changes between with and without ICG-NIR administration were observed. MMP was normalized to the mean of the values of controls and compared between groups by using analysis of variance. **(C)** Cell apoptosis was detected through the annexin V assay. The *x*-axis was the annexin V signal which represented the expression of phosphatidylserine on the membrane when cells underwent apoptosis. The *y*-axis was the PI signal which represented the loss of membrane integrity of cells undergoing necrosis. The lower left, upper left, lower right, and upper right portions respectively indicate viable, necrotic, apoptotic, and secondary necrotic cells. **(D)** Apoptosis-associated proteins were analyzed in OATP1B3-expresssing HT-1080 cells with different treatment. **(E)** Western blotting assays were done in triplicate, then all data were further quantified. Error bars indicate the SEM. *p < 0.05, **p < 0.01, and ***p < 0.001. The scale bar is 100 μm.

Several studies have reported that ROS causes oxidative damage in the mitochondria and affects their function (Guo et al., 2013; Murphy, 2013; Ježek et al., 2018). Thus, we looked into mitochondrial membrane potential (MMP) by treating the cells with the JC-1 dye to determine changes. The results revealed that ICG-NIR exposure promoted mitochondrial depolarization in the OATP1B3-expressing HT-1080 cells. Furthermore, ICG-NIR exerted a significantly stronger effect on mitochondrial depolarization than did control (Figure 3B). A decreased red (−590 nm) to green (−529 nm) fluorescence intensity ratio in response to ICG-NIR exposure for mitochondrial depolarization was noted in fluorescence images (Figure 3A). In addition, fluorescence images indicated that ICG-NIR irradiation induced higher ROS production within the OATP1B3-expressing HT-1080 cells and resulted in ROS accumulation in response to mitochondrial dysfunction. These results implied that the loss of MMP is a signal of stress and may release apoptotic factors, leading to cell death. To further understand ICG-NIR exposure impaired MMP and triggered apoptosis in OATP1B3-expressing HT-1080 cells, we examined cell apoptosis by using the annexin V assay. The number of apoptotic cells increased dramatically after ICG-NIR administration (Figure 3C). Moreover, the expression levels of cell apoptosis-related genes were also studied through Western blotting. The results showed an increase in cleaved PARP-1, cleaved Caspase-3, Bax and Bim but a decrease in Bcl-2 was observed under ICG-NIR irradiation (Figures 3D,E). In summary, our results implied that PDT treatment following of ROS accumulation attenuated MMP and induced cell death *via* apoptosis.

### 3.4 Photothermal assessment

**FIGURE 4:**
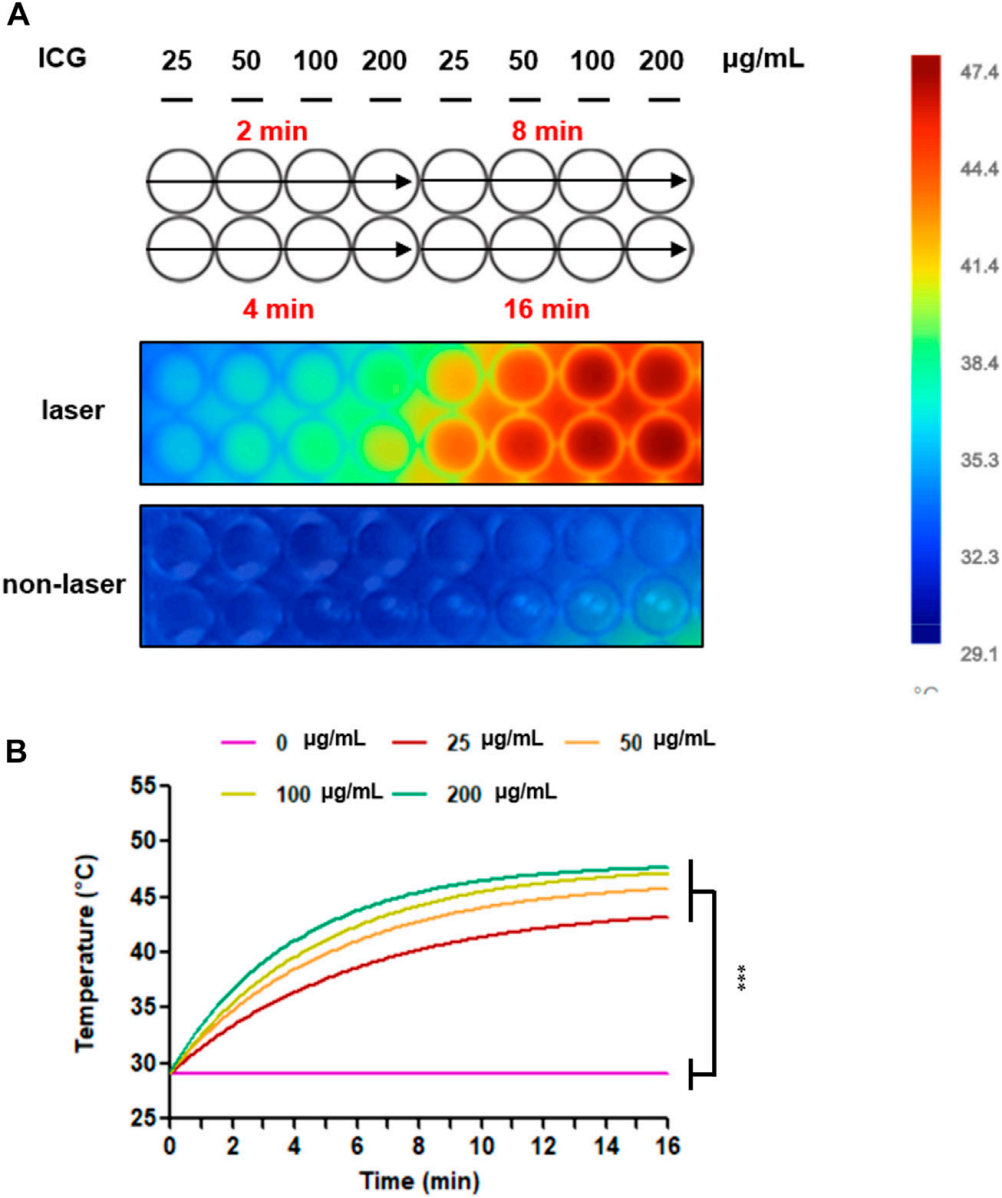
Temperature increases after treatment with ICG and 808-nm laser irradiation. **(A)** Changes in the thermal images of colors were examined *in vitro* by using an infrared thermal imaging camera. The bar displayed the temperature scale to distinguish the temperature variation. **(B)** The thermal heating curves were analyzed and quantified using Fluke Connect. Temperature increases between the laser and non-laser conditions. The data are presented as the mean ± SEM; *n* = 3 per group (***p < 0.001).

The photothermal heating capacity of ICG was evaluated in an aqueous dispersion. The temperature variation was recorded under continuous laser irradiation (1 W/cm^2^) for 2, 4, 8, and 16 min, respectively, after treatment with the ICG concentrations of 25, 50, 100, and 200 μg/mL. As depicted in Figure 4, the temperature was higher under the laser condition than under the non-laser condition. Furthermore, at the same laser power density, the temperature of the OATP1B3-expressing HT-1080 cells treated with 200 μg/ml ICG increased from 29.1 to 47.4°C; this finding is consistent with that of software analysis and quantification. Similar increases in temperature were noted in the remaining treatment groups (25, 50, and 100 μg/ml), indicating that the OATP1B3-expressing HT-1080 cells treated with ICG exhibited a favorable hyperthermic effect upon 808-nm laser irradiation.

### 3.5 Evaluation of cell viability after ICG and near-infrared laser irradiation

**FIGURE 5:**
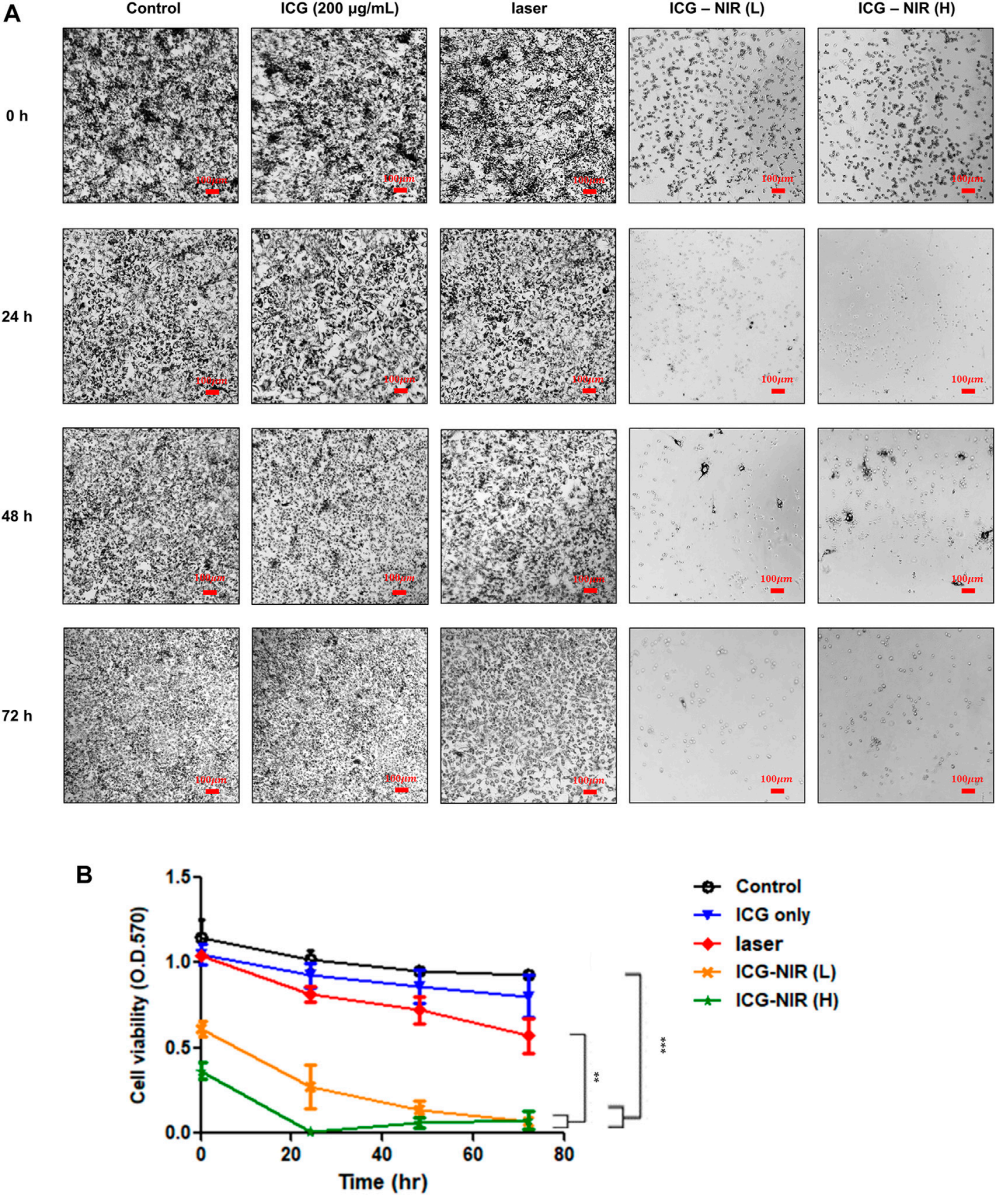
MTT metabolism of OATP1B3-expressing HT-1080 cells after ICG treatment and irradiation *in vitro*. **(A)** Cell viability was examined using the MTT assay. Morphological changes and formazan crystal formation in the OATP1B3-expressing HT-1080 cells were observed under a light microscope. Control, untreated cells; ICG, cells treated with ICG (200 μg/ml); Laser only, cells exposed to 808-nm NIR; ICG-NIR, cells treated with both ICG and 808-nm light exposure; L, cells exposed to low-fluence laser irradiation; H, cells exposed with high-fluence laser irradiation. **(B)** A significant difference was observed in cell viability between the ICG-NIR and control groups. Data represent the findings of three separate experiments, and values are presented as the mean ± standard error. ***p < 0.001. The scale bar is 100 μm.

To determine whether ICG-NIR treatment reduces the cell population and causes cell death, we performed the 3-[4,5-dimethylthiazol 2-yl]-2,5-diphenyl tetrazolium bromide (MTT) assay. Purple formazan crystals were formed only in live treated cells because of the presence of mitochondrial reductases. As presented in Figure 5A, the formation of formazan crystals in the ICG-NIR group was fewer than that in the control group, as observed under a light microscope. Moreover, we examined the absorbance of this colored solution at 570 nm (Figure 5B). The results revealed that fewer viable cells were present in the ICG-NIR group, indicating that the suppression of cell survival following ICG-NIR treatment was effective.

### 3.6 Ferroptosis occurred upon ICG-near-infrared irradiation

**FIGURE 6:**
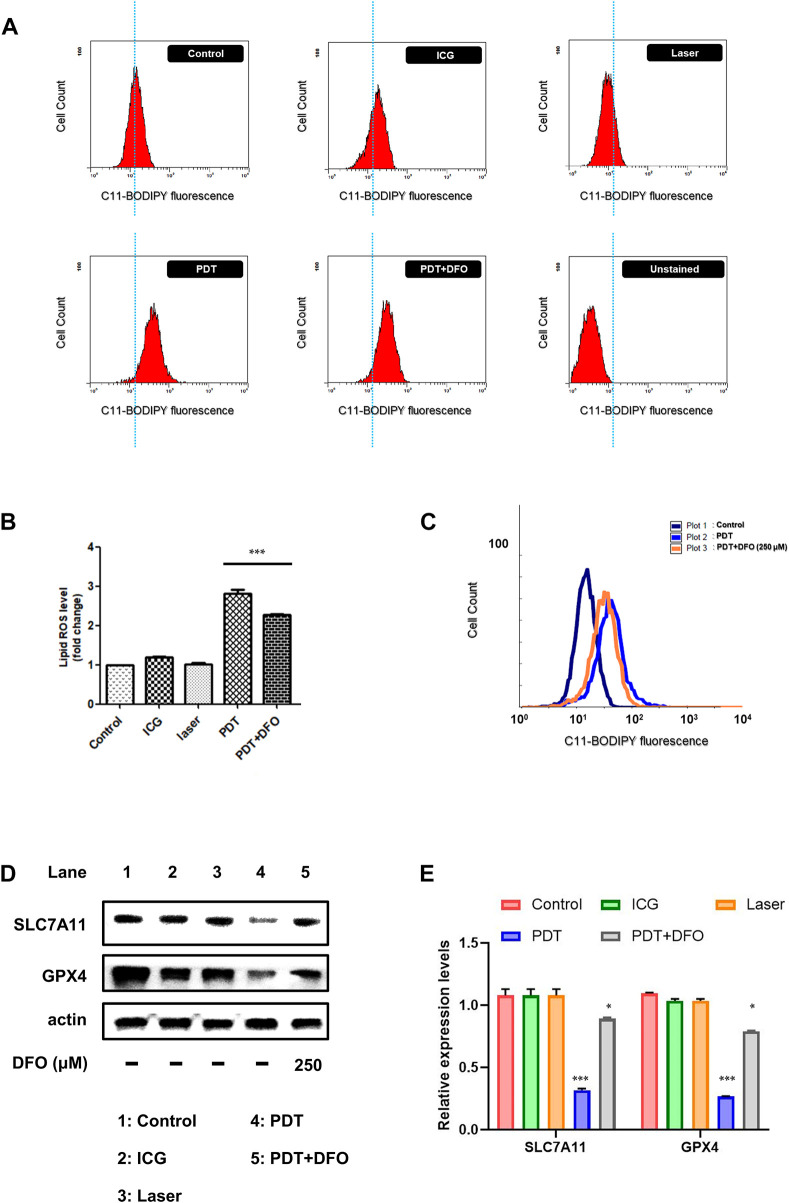
ICG-NIR irradiation induced cell death through the ferroptosis pathway. **(A)** Changes in the cellular lipid ROS levels of control (with C11-BODIPY staining), ICG (cells treated with 200 μg/ml ICG only), laser (cells treated with 808-nm laser only), PDT (cells incubated with ICG and then exposed to 808-nm laser), PDT + DFO (cells treated with PDT and 250 μM DFO), and unstained (cells without C11-BODIPY staining) groups. **(B)** Bar graph showing the mean fluorescence intensity of lipid ROS generation. **(C)** Representative flow cytometry histogram with an overlay of the three groups indicating that DFO alleviated ICG-NIR-induced ferroptosis. **(D)** Changes in the protein expression of SLC7A11 and GPX4. β-Actin was used as an internal control. **(E)** Western blotting assays were further quantified. Error bars represent the SEM from three independent replicates (*n* = 3). ***p < 0.001.

*In vitro* photodynamic therapy (PDT) caused the death of the OATP1B3-expressing HT-1080 cells; this finding is consistent with that of the cell viability assay (Figure 5). To elucidate whether other mechanisms through which ICG-NIR irradiation affects cell survival, we reviewed previous studies (Wang et al., 2019b; Chen et al., 2022). The OATP1B3-expressing cells treated with ICG-NIR may have a higher tendency to undergo ferroptosis owing to the overall higher active metabolism and higher ROS load. We first measured the lipid ROS level to verify our hypothesis because studies have demonstrated that ferroptosis results from lipid peroxidation (Zuo et al., 2020; Jiang et al., 2021). The results of flow cytometry (Figure 6A) indicated that the lipid ROS level was higher in the PDT group than in either the control, ICG, or laser group. We then performed Western blotting to examine the protein expression of SLC7A11 and GPX4 because both of these proteins were reported to serve as biomarkers for ferroptosis (Jiang et al., 2021). When SLC7A11 and GPX4 are dysfunctional, lipid transforms into lipid ROS with O_2_ and Fe^2+^, resulting in lipid ROS attacking intracellular biomolecules and finally killing cells. Our results indicated that the expression of SLC7A11 and GPX4 was downregulated (Figure 6D, lane 1 vs. lane 4 and Figure 6E) in the PDT group compared with the ICG and laser groups. In addition, we examined if ICG-NIR irradiation leads to ferroptosis by using deferoxamine mesylate salt (DFO, an iron chelator), a ferroptosis inhibitor, in this study. DFO could directly regulate iron metabolism to inhibit ferroptosis. As presented in Figures 6B,C, the lipid ROS level was attenuated in the presence of DFO. Furthermore, DFO reversed the downregulation of GPX4 and SLC7A11 induced by ICG-NIR (Figure 6D, lane 5 and 6e). The findings suggested that the OATP1B3-expressing cells die upon ICG-NIR administration at least partially through the ferroptosis pathway.

### 3.7 *In vivo* PDT of nude mice with subcutaneous tumors

**FIGURE 7:**
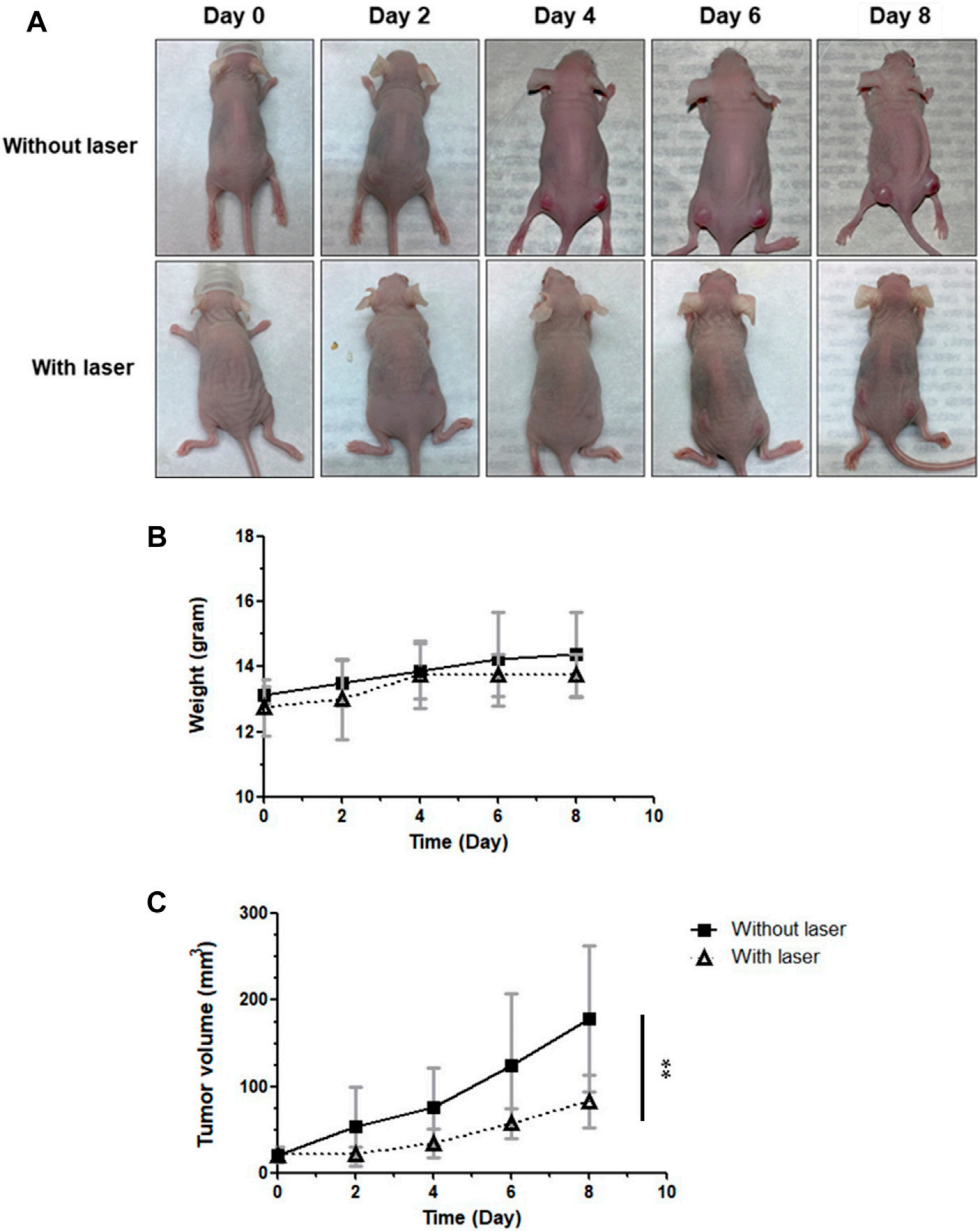
Effect of PDT on antitumor capabilities *in vivo*. ICG was administered every day following laser irradiation. The tumor volumes and body weights were measured at indicated times. **(A)** Representative photos of HT-1080-OATP1B3-bearing mice receiving PDT. **(B)** Body weights of mice after treatment. **(C)** Relative tumor change curves during 808-nm laser irradiation. The results are presented as the mean ± SEM (*n* = 8 per group, **p < 0.01 between the laser and non-laser groups.).

On the basis of the findings of ICG-NIR treatment *in vitro*, we investigated the effects of tumor therapy *in vivo*. The mice were divided into two groups: group 1 (control; ICG treatment without laser irradiation) and group 2 (PDT; ICG treatment with laser irradiation). On the basis of IVIS results presented in Supplementary Figure S3, we applied laser treatment to the tumor 24 h after the administration of ICG. After conducting different therapies, tumor volumes were measured every 2 days. Finally, we sacrificed mice for obtaining tumors. Specimens were collected, and lipid peroxidation was examined through immunohistochemical (IHC) staining (Supplementary Figure S4). During the treatment, no marked difference in weight changes was noted among the groups, suggesting minimal damage by PDT (Figure 7B). However, as presented in Figures 7A,C marked difference in tumor volumes was observed between the groups receiving ICG without laser irradiation and ICG with laser irradiation, implying that ICG-NIR exerted a tumor inhibitory effect on the OATP1B3-expressing cells. The digital photos of mice in distinct groups were obtained to illustrate the therapeutic effect visually (Figure 7A).

## 4 Discussion

This study demonstrated the multiple anticancer properties of ICG. ICG exhibited photothermal and photodynamic effects, eventually causing apoptosis and ferroptosis. Multimodal and combination cancer therapies have changed the landscape of anticancer treatment (Meric-Bernstam et al., 2021; Gugenheim et al., 2022). Various medications focusing on different cancer-specific pathways increase patient survival. ICG can be used in multimodal or combination anticancer therapy for several reasons. First, ICG has been clinically used for decades. Its broad therapeutic range and safety were reported (Lu and Hsiao, 2021). Second, because ICG is stimulated at the infrared activation range, it may penetrate deeper into cancer tissues where visible light cannot reach. Third, we previously observed that the metabolism of ICG is regulated by several transmembrane proteins such as the OATP family. Moreover, OATP proteins are expressed in several cancer cells (Thakkar et al., 2013; Morio et al., 2018; Chen et al., 2020), making ICG a potential theranostic molecule.

A relationship exists between OATP1B3 and cancers. OATP1B3 is highly expressed in breast, gastrointestinal tract, lung, prostate, colon, and pancreatic cancers (Abe et al., 2001; Monks et al., 2007; Muto et al., 2007; Hamada et al., 2008; Lee et al., 2008; Kounnis et al., 2011). Furthermore, OATP1B3 protein expression is positively correlated with the clinicopathological features of patients with cancer and has a predictive value. Hence, examining ICG-treated OATP1B3-expressing cells exposed to laser irradiation might facilitate the development of cancer therapies. This study proposed that the elevated ROS level and photothermal effect mediated the cell death of the OATP1B3-expressing cells after ICG-NIR administration. These findings have been reported previously (Sheng et al., 2018), indicating that OATP1B3-expressing cells can be a significant target during PDT because they have a more substantial capacity of intaking ICG. Although other studies have indicated that oxidative stress, hyperthermic effect, and repeated NIR laser exposure play essential roles in the effectiveness of PDT (Dolmans et al., 2003; Brown et al., 2004; Wu et al., 2020), our findings indicated that the quenching effect of ICG may be another critical factor underlying the anti-tumor effect of ICG-NIR therapy due to higher ROS production.

ICG strongly absorbs NIR irradiation; thus, it can convert absorbed light energy into ROS and heat. This study examined mechanisms through which ICG-NIR treatment causes ROS production and heat generation in OATP1B3-expressing cells. Our results indicated that high ROS accumulation in cells led to a decrease in MMP, followed by the initiation of apoptosis (Ly et al., 2003). In addition, the OATP1B3-expressing cells became more spherical, and cell viability decreased and cell death increased in a time-dependent manner. Similar results or tendencies have been demonstrated in other studies examining the effects of photosensitizers on various cell lines (Zhang et al., 2019; Jiang et al., 2020; Park et al., 2020).

Our results also indicated that ICG-NIR administration caused death in the OATP1B3-expressing cells through ferroptosis. This unique modality of cell death, driven by iron-dependent phospholipid peroxidation, is regulated by multiple cellular metabolic pathways. Accumulating research has implied that many cancer cells show increased susceptibility to ferroptosis, and ferroptosis induction can be explored as an anticancer therapy (Zuo et al., 2020; Jiang et al., 2021). However, this newly discovered form of cell death and its connection with cancer should be examined. Essential regulators of ferroptosis, such as a crucial inhibitor of phospholipid peroxidation, glutathione peroxidase 4 (GPX4), and the system x_c_^−^ cystine/glutamate antiporter (a transmembrane protein complex containing subunits SLC7A11 and SLC3A2), inhibit ferroptosis by contributing to GPX4 activity. Our findings suggested that ICG-NIR treatment induced ferroptosis through the regulation of GPX4 and SLC7A11 and the accumulation of lipid ROS, as indicated by the results of the Western blot and C11-BODIPY fluorescence staining (Figure 6) in the OATP1B3-expressing cells.

Given its optical absorbance in the NIR region, ICG has been widely used in fluorescence imaging. *In vivo* imaging offers a unique approach to visualizing the dynamic distribution of ICG within the whole body. Our imaging data demonstrated that ICG was incorporated explicitly into and visualized in the subcutaneous xenograft OATP1B3-expressing tumors of mice 24 h post intraperitoneal injection (Supplementary Figure S3A). The background uptake in most tissues has been low because OATP1B3 exhibits restricted tissue expression (Svoboda et al., 2011). Moreover, PDT induced ROS production and suppressed tumor growth *in vivo* (Supplementary Figure S4 and Figure 7B). The 4HNE antibody is a promising antigen because it can be targeted to evaluate cellular ROS levels in tissues through IHC (Hu et al., 2020). Thus, we used 4HNE to detect oxidative stress–mediated lipid peroxidation. The IHC results indicated that OATP1B3-expressing tumors had a significantly high readout for ROS after ICG-NIR therapy.

Compared with other studies, the anti-tumor effect of PDT combined with ICG in our model mice was stronger because of the sufficient accumulation of ICG in OATP1B3-expressing tumors. The inadequate accumulation of ICG in tumors would adversely affect tumor growth prevention. Nevertheless, this study has some limitations. First, for measuring tumor sizes, our method should be improved because of the presence of measurement errors and anthropogenic factors. In the future, measurements conducted through imaging can synergistically provide information to delineate tumor size, scope, and guiding therapy accurately. In addition, the size of tumors is the major factor limiting the application of the findings of the present study to clinical practice. ICG-NIR therapy was found to be effective only for small tumors. Therefore, ICG-NIR treatment combined with other therapeutic tools may be necessary for clinical use. As for *in vivo* cell line-derived xenograft, we only compared the NIR effect of OATP1B3-expressing HT-1080 cells after ICG injection, we didn’t perform the comparison between HT-1080 and OATP1B3-expressing HT-1080 cells. The reason is that there is endogenous uptake of ICG of HT-1080 cells that might interfere with our interpretation of the ICG effect (Wu et al., 2018). However, the endogenous uptake of ICG in many cancer cells could be used as an advantage in future ICG-NIR irradiation treatment. Further study regarding the role of endogenous ICG in these cancer cells and the possibility of treatment through the ferroptosis pathway should be investigated.

Our findings have several implications for cancer treatment. With the progress in optics and robotic mechanics, more effective surgical instruments have been increasingly used in tumor surgery. In combination with these techniques, ICG has been used by surgeons to visualize undissected, residual tumors (Kaplan-Marans et al., 2019; Baiocchi et al., 2021). These tumors could be ablated by infrared laser through the aforementioned mechanisms. Additionally, ICG can be easily encapsulated into nanocomposites as a small molecule, making the implementation of a cancer-specific, multiple functionality theranostic strategy possible (Wang et al., 2017; Wang et al., 2019a). Finally, for cancer cells exhibiting anti-ferroptotic mechanisms or expressing cancer-type OATP1B3, our findings on the ferroptotic effect of ICG and ICG metabolism might contribute to eradication of cancer cells.

## 5 Conclusion

**FIGURE 8:**
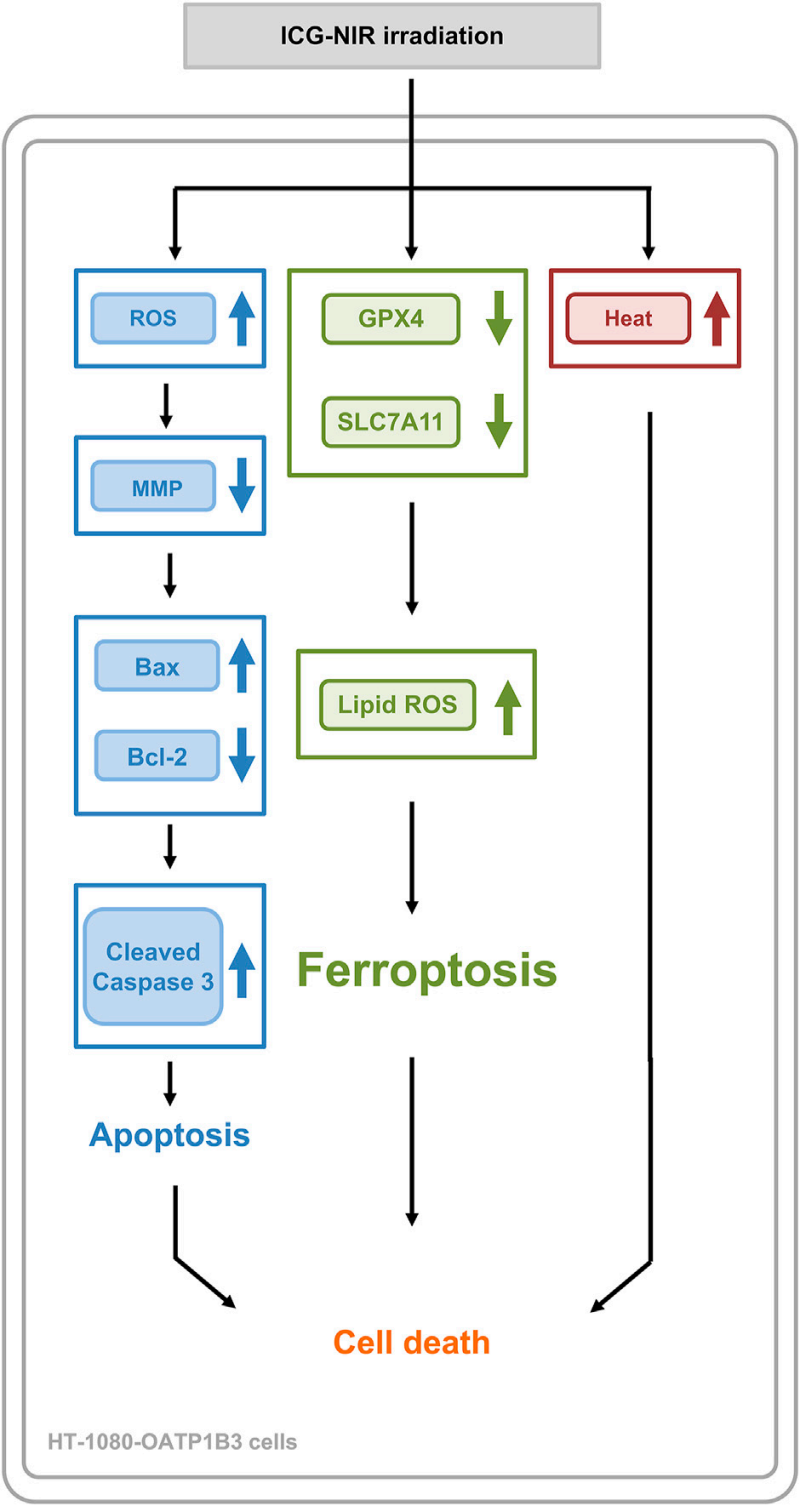
A summary diagram outlining the working mechanism of ICG-NIR irradiation in HT-1080-OATP1B3 cells. ICG-NIR irradiation increased heat and ROS production following a marked decrease in MMP. In addition, ICG-NIR irradiation triggered apoptosis and induced ferroptosis through the downregulation of GPX4 and SLC7A11 as well as lipid ROS overproduction.

In addition to the photodynamic and photothermal effect, ICG with NIR irradiation successfully enhanced the death of the OATP1B3-expressing cells through apoptosis and ferroptosis. Furthermore, the quenching effect of ICG may be a crucial factor in effective PDT due to the higher production of ROS and the severe loss of MMP. The multiple mechanisms of cancer therapy (Figure 8) in a single molecule can provide conceptual and mechanistic insights into cancer theranostic strategy.

## Data Availability

The original contributions presented in the study are included in the article/Supplementary Material, further inquiries can be directed to the corresponding author.

## Supplementary material

SUPPLEMENTARY FIGURE S1 HT-1080 OATP1B3-expressing cells after ICG incubation.

SUPPLEMENTARY FIGURE S2 Schematic diagram of the NIR laser source for *in vitro* and *in vivo* PDT.

SUPPLEMENTARY FIGURE S3
*In vivo* fluorescence imaging in ICG-treated mice with subcutaneous tumors.

SUPPLEMENTARY FIGURE S4 Histological analysis of subcutaneous tumors in ICG-NIR treated mice.

## Abbreviations

BSA, bovine serum albumin; BCA, bicinchoninic acid; CCCP, carbonyl cyanide 3-chlorophenylhydrazone; DMEM, dulbecco’s modified eagle’s medium; DCFH-DA, 2′,7′-dichlorofluorescein diacetate; DAPI, 4,6-diamidino-2-phenylindole; DFO, deferoxamine mesylate salt; FBS, fetal bovine serum; GPX4, glutathione peroxidase 4; ICG, indocyanine green; IHC, immunohistochemistry; IACUC, institutional animal care and use committee; MMP, mitochondrial membrane potential; MTT, 3-[4,5-dimethylthiazol 2-yl]-2,5-diphenyl tetrazolium bromide; NIR, near-infrared; OATP, organic anion transmembrane polypeptide; PDT, photodynamic therapy; PBS, phosphate-buffered saline; PBST, phosphate-buffered saline with Tween-20; PI, propidium iodide; ROS, reactive oxygen species; ROI, region of interest; SLC7A11, a subunit of the system xc−cystine/glutamate antiporter.
