# Supplementary material for: Indocyanine green as a near-infrared theranostic agent for ferroptosis and apoptosis-based, photothermal, and photodynamic cancer therapy
Source: Front Mol Biosci. 2022 Dec 7;9:1045885. doi: 10.3389/fmolb.2022.1045885 (PMC9768228; doi:10.3389/fmolb.2022.1045885)
Supplement: Supplementary file 1 [file DataSheet1.docx]

Figure S1


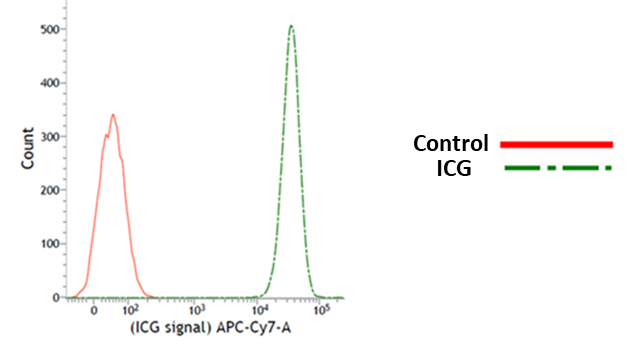


Figure S1. HT-1080 OATP1B3-expressing cells after ICG incubation. The cells were analyzed by using flow cytometry at the APC-Cy7 wavelength to determine the intensity of ICG. Control indicated in red color represented cells without adding ICG; ICG indicated in green color represented cells with ICG treatment.

Figure S2

**A**

**
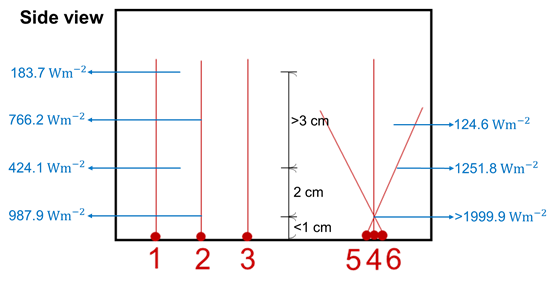
**

**B**

**
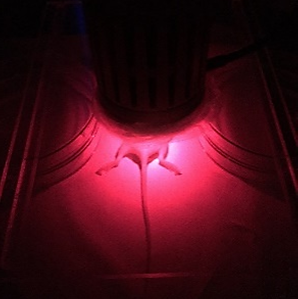
**

Figure S2. Schematic diagram of the NIR laser source for *in vitro* and *in vivo* PDT. (A) The fluence rate of our homemade laser source for *in vitro* PDT was measured by LI-250A Light Meter at distinct vertical height. (B) The fluence rate of High Power LED Driver (DC2200) for *in vivo* PDT was 1 W/cm^2^. The device was manufactured by Thorlabs GmbH, Münchner, Germany.

Figure S3

**A B**

**
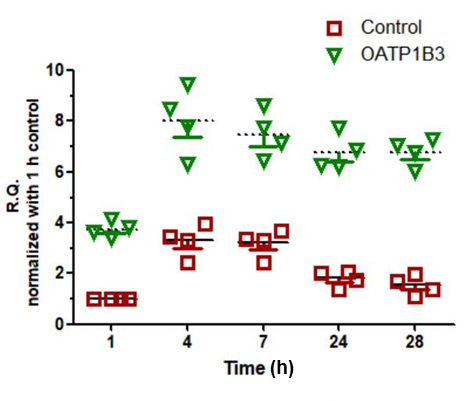

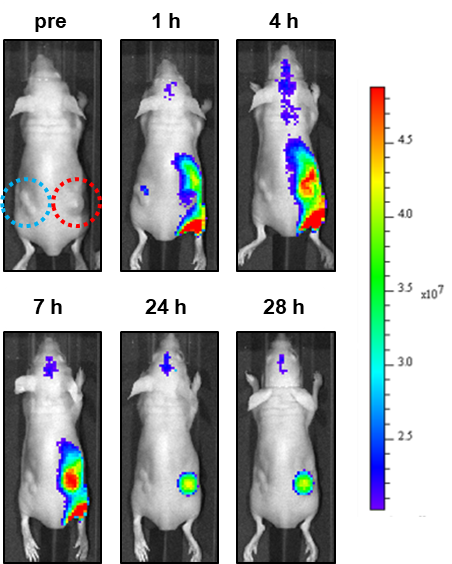
**

Figure S3. *In vivo* fluorescence imaging in ICG-treated mice with subcutaneous tumors. (A) After implanting OATP1B3-expressing cells, we administered 10 mg/kg of ICG. Next, we tracked the ICG signal from 1 to 28 h. The blue- and red-dotted circle indicate the control tumor and OATP1B3-expressing tumor, respectively. (B) The bar graph showed the IVIS relative quantification normalized to the region of interest (ROI) of the 1 h control tumor. The ROI data were acquired from the dotted circles (n = 4). The ROI of the OATP1B3 tumor was compared with that of the control at each time point. R.Q., relative quantitation.

Figure S4

**
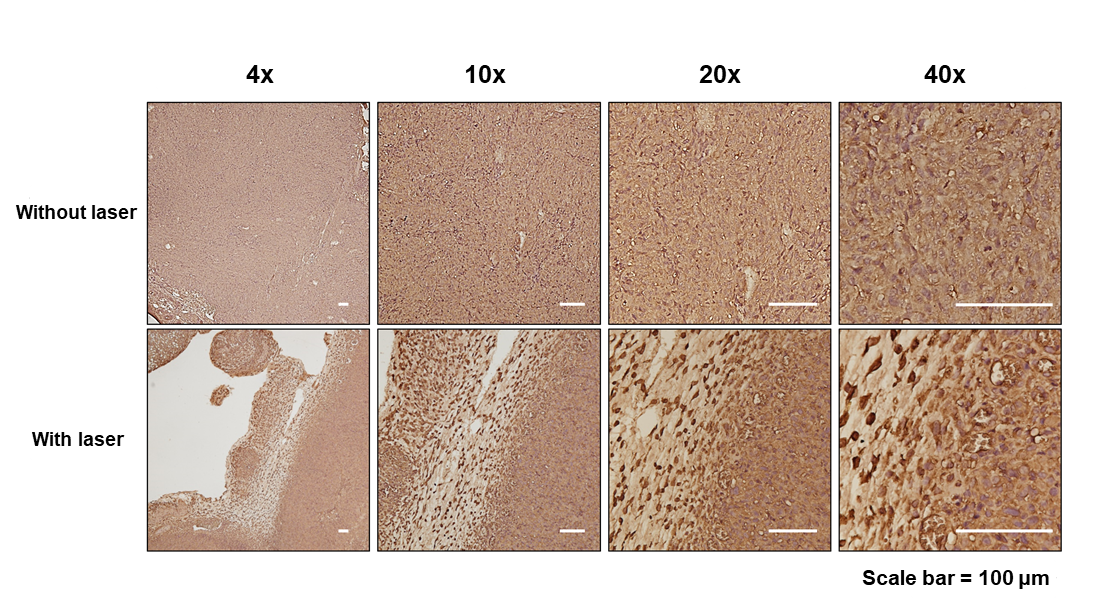
**

Figure S4. Histological analysis of subcutaneous tumors in ICG-NIR treated mice. IHC stained results of tumor sections that were collected from mice of different treatments. Brown color indicated detection of oxidative stress-mediated lipid peroxidation. The scale bar is 100 μm.
